# 

**Published:** 1910-10-15

**Authors:** 


### ANNOUNCEMENTS.

### MICHIGAN STATE BOARD OF EXAMINERS.

The next regular meeting of the Michigan State Board of Dental Examiners will be held at Ann Arbor, Nov. 16 to 19 inclusive.

A. W. Haidle, *Sec'y*,
Negaunee, Mich.

### OHIO STATE DENTAL SOCIETY.

The Forty-Fifth annual session of the Ohio State Dental Society will be held in Columbus on Dec. 6, 7 and 8, 1910.

The Great Southern Hotel will be headquarters as heretofore, but additional space will be available giving more room for the meetings of the society and the clinics which of late years have been so much overcrowded.

Come and hear a Symposium on the Physiology and Pathology of the Sockets and Gums by men of wide reputation.

Dr. M. H. Fletcher, the President, on "Reparative powers of Cementum".

"Physiology and Anatomy of the Gums and Sockets," by an essayist yet to be secured.

Dr. T. B. Hartzell, of Minneapolis, On "Infection and Pathology of the Gums and Sockets."

Dr. N. S. Hoff, of Ann Arbor, on "Instrumentation and Medication of Gums and Sockets."

Dr. Joseph Head, of Philadelphia, on "Prophylaxis of Gums and Sockets."

Dr. S. L. McCurdy, of Pittsburg, on "Extended Diseases of the Sockets and Bones; Surgical Treatment."

These papers, with clinics along the same line, will afford an invaluable course of instruction in the cause and treatment of these diseases.

The numerous other clinics, covering the entire range of dental practice, will contain many features of value to every member.

Mark the dates on your appointment book now, and come to another of those big meetings of the Ohio State Dental Society.

F. R. Chapman, *Secretary.*

### NEW YORK SEVENTH DISTRICT DENTAL SOCIETY.

The Annual Union Meeting of the 7th and 8th Dist. Dental Societies of the State of New York will be held at Rochester in the Hotel Seneca on Nov. 10, 11 and 12th, 1910.

An especially good programme has been prepared. All ethical dentists are invited to attend these meetings.

George C. Lowe, *Rec. Sec'y.*

